# Proptosis with Increased Orbital Fat in an Obese Patient

**DOI:** 10.1155/2020/8832704

**Published:** 2020-12-08

**Authors:** Azhar Seedat, Shaheen Seedat, Sulaiman E. I. Moosa

**Affiliations:** ^1^Division of Radiology, Department of Radiation Medicine, Groote Schuur Hospital and University of Cape Town, Cape Town, South Africa; ^2^Department of Health Care Policy and Research, Weill Cornell Medicine, Cornell University, New York City, New York, USA; ^3^Infectious Disease Epidemiology Group, Weill Cornell Medical College, Cornell University, Doha, Qatar

## Abstract

Computational tomography (CT) is a well-documented modality in the workup of proptosis. We present a case of proptosis due to increased orbital fat in an obese patient. We review the literature to discuss the most likely causes of increased orbital fat, and we discuss the utility of CT imaging in assessing this pathology.

## 1. Introduction

Proptosis is the abnormal forward protrusion of the globe from the orbit. CT imaging is useful both to diagnose and quantify the degree of protrusion.

## 2. Case Presentation

A 51-year-old male known with diabetes, hypertension, ischemic heart disease, chronic kidney disease, upper gastrointestinal bleeding, and morbid obesity was referred for a CT brain by the ophthalmology department as part of the workup of proptosis. The patient had no clinical features to suggest Graves' disease or Cushing's syndrome. The TSH (15.3 pmol/L) as well as the 08:00 serum cortisol (345 nmol/L) were normal.

**Figure 1:**
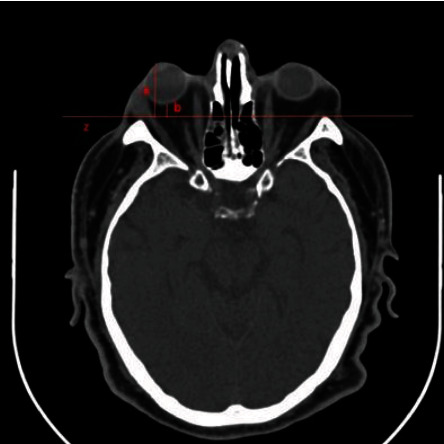
Axial CT brain at the level of the optic nerve head. Note the prominence of retroorbital fat. z: interzygomatic line; a: distance to the anterior sclera is 29.9 mm; b: posterior sclera is 6.3 mm anterior to the interzygomatic line.

The CT brain showed bilateral proptosis measuring 29.9 mm from the anterior aspect of the globe to the interzygomatic line, and the posterior aspect of the globe was 6.3 mm anterior to the interzygomatic line (Figure 1). Marked prominence of retroorbital fat was evident. The globe, extraocular muscles, skeletal structure, and vessels were normal. Importantly, there were no ocular, intraconal, or extraconal masses. The rest of the intracranial structures, including the pituitary gland, were normal with no features of hydrocephalus.

## 3. Discussion

Proptosis is the abnormal protrusion of the globe and can be measured accurately with CT. In 1989, Gibson established a reliable and reproducible method of measuring proptosis using CT [1]. In order to measure the anterior protrusion of the globe, a line is drawn across the zygomatic bone, known as the interzygomatic line. The normal reference values are as follows: the posterior aspect of the sclera is 9.9 mm ± 1.7 mm posterior to the interzygomatic line, and the anterior aspect of the sclera is less than 23 mm from the interzygomatic line [2, 3].

The differential diagnosis for increased retroorbital fat primarily includes Graves' disease, Cushing's disease/syndrome, and obesity.

Graves' disease is the most common cause of proptosis. It can frequently cause an increase in orbital fat volume giving it a “dirty” appearance [4]. It may also result in a spindle-shaped enlargement of the extraocular muscles (most frequently the inferior rectus) sparing the tendons [4, 5] as well as an increase in the bony orbital volume [6]. Graves' ophthalmopathy typically causes extraocular muscle enlargement early on, followed by an increase in orbital fat. In a small subset of patients, increase in orbital fat can occur independently [7]. However, whilst Potgieser and colleagues also agree that over time Graves' disease causes an increase in orbital fat volume, they found a progressive decrease in muscle volume over time [8].

Despite Harvey Cushing first describing proptosis in a patient with Cushing's disease in 1932, it is an often overlooked cause [9, 10]. Proptosis can occur in 30-45% of patients with Cushing's disease [10]. It is important to note that proptosis can be associated with other causes of Cushing's syndrome which include iatrogenic causes such as systemic steroids [11, 12], adrenal gland pathologies [9], and tumors outside the pituitary-adrenal axis, e.g., small-cell lung cancer and CRH-secreting tumors. Other causes include postretrobulbar corticosteroid injection causing local fat proliferation [13].

On CT scan, patients with Cushing's disease have an increase in orbital fat volume, and extraocular muscles are unaffected [14]. When reporting on proptosis, it is imperative to evaluate the pituitary gland as enlargement is suggestive of a pituitary adenoma.

With regard to proptosis secondary to obesity, Peyster et al. demonstrated the relationship between general body fat, orbital fat volume, and proptosis [14]. The study looked at CT scans of fifteen patients with proptosis as a result of increased orbital fat. Four patients had Graves' disease, two patients had Cushing's disease or syndrome, and nine patients were obese with no endocrinopathy. The scans were compared to a control group of sixteen patients without proptosis. They were able to show a strong positive association between orbital fat volume and proptosis for all patients (*r* = 0.76, *p* < 0.001). Furthermore, they showed that the correlation coefficient between orbital fat volume and proptosis was even larger (*r* = 0.79) in the group of patients with obesity and without endocrinopathy (Graves' disease and Cushing's syndrome).

In addition, Schmidt and colleagues used MRI to demonstrate the positive correlation between body mass index (BMI)/waist circumference and the degree of proptosis (*p* < 0.001) [15].

## 4. Conclusion

Studies have shown that Graves' disease, Cushing's syndrome, and obesity are the most likely causes of increased orbital fat causing proptosis. Reported case studies focusing exclusively on increased orbital fat secondary to obesity are limited. Given that our patient did not have either Graves' or Cushing's disease/syndrome and considering available literature, we present a case of marked proptosis due to obesity. It is important for the radiologist to be aware of obesity as a cause of proptosis.
